# A multi‐institutional randomized controlled trial comparing first‐generation transrectal high‐resolution micro‐ultrasound with conventional frequency transrectal ultrasound for prostate biopsy

**DOI:** 10.1002/bco2.59

**Published:** 2020-11-28

**Authors:** C. P. Pavlovich, M. E. Hyndman, G. Eure, S. Ghai, Y. Caumartin, E. Herget, J. D. Young, D. Wiseman, C. Caughlin, R. Gray, S. Wason, L. Mettee, M. Lodde, A. Toi, T. Dujardin, R. Lance, S. M. Schatz, M. Fabrizio, J. B. Malcolm, V. Fradet

**Affiliations:** ^1^ The Brady Urological Institute The Johns Hopkins School of Medicine Baltimore MD USA; ^2^ Southern Alberta Institute of Urology and Prostate Cancer Centre Calgary AB Canada; ^3^ Urology of Virginia Department of Urology Eastern Virginia Medical School Virginia Beach VA USA; ^4^ Joint Department of Medical imaging University Health Network University of Toronto Toronto ON Canada; ^5^ Centre de Recherche en Cancérologie de l’Université Laval Quebec City QC Canada; ^6^ Houston Methodist Institute for Academic Medicine Houston TX USA

**Keywords:** micro‐ultrasound, high‐frequency ultrasound, prostate cancer, prostate biopsy, PRI‐MUS, ExactVu

## Abstract

**Objectives:**

To study high‐frequency 29 MHz transrectal side‐fire micro‐ultrasound (micro‐US) for the detection of clinically significant prostate cancer (csPCa) on prostate biopsy, and validate an image interpretation protocol for micro‐US imaging of the prostate.

**Materials and methods:**

A prospective randomized clinical trial was performed where 1676 men with indications for prostate biopsy and without known prostate cancer were randomized 1:1 to micro‐US vs conventional end‐fire ultrasound (conv‐US) transrectal‐guided prostate biopsy across five sites in North America. The trial was split into two phases, before and after training on a micro‐US image interpretation protocol that was developed during the trial using data from the pre‐training micro‐US arm. Investigators received a standardized training program mid‐trial, and the post‐training micro‐US data were used to examine the training effect.

**Results:**

Detection of csPCa (the primary outcome) was no better with the first‐generation micro‐US system than with conv‐US in the overall population (34.6% vs 36.6%, respectively, *P* = .21). Data from the first portion of the trial were, however, used to develop an image interpretation protocol termed PRI‐MUS in order to address the lack of understanding of the appearance of cancer under micro‐US. Micro‐US sensitivity in the post‐training group improved to 60.8% from 24.6% (*P *< .01), while specificity decreased (from 84.2% to 63.2%). Detection of csPCa in the micro‐US arm increased by 7% after training (32% to 39%, *P* < .03), but training instituted mid‐trial did not affect the overall results of the comparison between arms.

**Conclusion:**

Micro‐US provided no clear benefit over conv‐US for the detection of csPCa at biopsy. However, it became evident during the trial that training and increasing experience with this novel technology improved the performance of this first‐generation system.

## Abstract 2

In this work high‐frequency 29 MHz transrectal side‐fire micro‐ultrasound (US) for the detection of clinically significant prostate cancer on prostate biopsy is studied, and an image interpretation protocol for micro‐US imaging of the prostate is validated.

The YouTube link is here: https://youtu.be/U2Svj-4Ae_k

## 1 INTRODUCTION

Transrectal ultrasound (TRUS) is the standard for prostate imaging during needle biopsy. Conventional TRUS frequencies of 5‐12 MHz provide good visualization of the prostate contour and some zonal anatomy but perform inadequately for prostate cancer detection. Generally, TRUS is used only to direct systematic sampling of the prostate during biopsy.^1^, ^2^ Despite its inadequacies, including limited resolution and difficulty in differentiating cancer from benign prostatic conditions, conventional TRUS has proven difficult to improve upon. While there have been advances in functional ultrasound, including power Doppler, tissue elastography, and contrast administration, none has entered the standard of care, in part due to operator dependency and modest sensitivity improvements over conventional gray‐scale B‐mode TRUS.

The most significant recent advance in prostate imaging has come not from ultrasound but from magnetic resonance imaging (MRI). Prostate MRI provides good discriminative ability to identify large and/or high‐grade lesions.^3^, ^4^ Multiparametric MR imaging of the prostate (mpMRI) and use of the PI‐RADS reporting system are altering the algorithm for prostate cancer detection.^5^ While mpMRI was first proposed in 2000,^6^ it did not gain significant traction outside of academic centers until a standardized interpretation and reporting protocol (PI‐RADS) was released by an expert ESUR committee in 2012.^7^ Even now, inter‐reader variability remains a concern and updates to the protocol have attempted to improve consistency.^8^ Nevertheless, mpMRI remains poor at detecting smaller high‐grade, and even larger low‐grade lesions, has issues with specificity overall, and has limitations for prostate cancer active surveillance.^9^, ^10^ MRI‐fusion or cognitive‐fusion to low‐resolution real‐time ultrasound images to guide biopsies is used when suspicious targets are detected, but a limitation here remains the difficulty of visualizing the lesion on ultrasound. Technical improvements in TRUS could, therefore, benefit both *de novo* prostate imaging as well as MRI‐fusion biopsy, and address some of the current limitations of prostate mpMRI and targeted biopsy (eg, cost, time, and low specificity).^11^, ^12^

An improved ultrasound visualization modality could potentially result in better detection rates of csPCa on initial biopsy, fewer MRIs, and inexpensive monitoring of men on active surveillance. To this end, when higher resolution 17‐29 MHz micro‐ultrasound (micro‐US) became available, a pilot study demonstrated improved prostate cancer visualization at these frequencies vs conventional TRUS.^13^ After technical improvements in probe design, micro‐US software and hardware, a side‐fire 512‐element micro‐US probe with a biopsy channel was created. We present here a multi‐institutional randomized controlled trial of TRUS‐guided prostate biopsy using either novel micro‐US or conventional ultrasound (conv‐US) in men with indication(s) for prostate biopsy. Pre‐specified in the trial plan was the development of an image interpretation protocol for micro‐US images that would define criteria for suspicious lesions on micro‐US.

## 2 MATERIALS AND METHODS

Five North American sites participated in this randomized clinical trial: Johns Hopkins University School of Medicine (Baltimore, USA), Southern Alberta Institute of Urology and Prostate Cancer Centre (Calgary, Canada), Princess Margaret Cancer Center (Toronto, Canada), the Centre de Recherche en Cancérologie de l'Université Laval (Quebec City, Canada), and Urology of Virginia/Eastern Virginia Medical School (Virginia Beach, USA). The trial enrolled 1676 men with indications for prostate biopsy (rising PSA, elevated PSA, and/or abnormal DRE) from 2013 to 2016. Investigators were experienced clinicians involved in prostate biopsy at each site and included Urologists and Uroradiologists. Funding was provided by Exact Imaging. Internal Review Board approval was provided by individual site ethics boards. The ClinicalTrials.gov registration number is NCT02079025.

### 2.1 Trial design

The trial design has been reported previously,^14^ but is summarized here briefly. Subjects were randomized 1:1 to one of two biopsy modalities: conventional ultrasound (conv‐US, using local standard of care TRUS systems) or micro‐ultrasound (micro‐US, using Exact Imaging’s first‐generation ExactVu™ system). The trial included men ages 40‐79, with PSA levels <50 ng/mL, and clinical stage <T3. Exclusion criteria were any history of prostate cancer, and men with known prostate volume (from any prior imaging) of >60cc. The trial was registered at clinicaltrials.gov as NCT02079025.

### 2.2 Outcomes

The pre‐specified primary outcome was the per‐patient detection of clinically significant prostate cancer (csPCa‐defined as Gleason Grade Group 2 or higher, and/or >50% cancer in any core) in each group. Pre‐specified secondary outcome measures were the effect of mid‐trial training (comparing pre‐ and post‐training micro‐US groups), and the performance characteristics of targeting in each arm of the trial on a per‐core level.

### 2.3 Statistical analysis

All detection rate variables were modeled as binomial random variables with beta (1,1) priors. These priors were updated to calculate the posterior probability density function given the observed data. These posterior probabilities were used to calculate confidence intervals, and *P*‐values for comparisons.

### 2.4 Mid‐trial training

Investigators were initially trained on the operation of the first‐generation side‐fire micro‐US device but without specific image interpretation training (given the lack of an image library of this new technology), each completing 3‐10 training cases before trial accrual. Later, the trial was paused after 1113 subjects (66.4%) in order to instruct investigators on lesion identification with micro‐US. This was done based on retrospective analyses of micro‐US cine‐loops and images acquired during the initial portion of this study correlated with csPCa found on histopathologic sections. The ultrasonographic appearance of these areas on micro‐US were studied, and a 5‐point scoring system for suspicious lesions was created, termed PRI‐MUS^TM^ (Prostate Risk Identification for Micro‐UltraSound).^15^ The PRI‐MUS^TM^ protocol was then used following the retraining hiatus by all investigators to score and target suspicious areas noted during TRUS‐biopsy in patients randomized to the micro‐US arm.

### 2.5 Biopsy protocol

**FIGURE 1:**
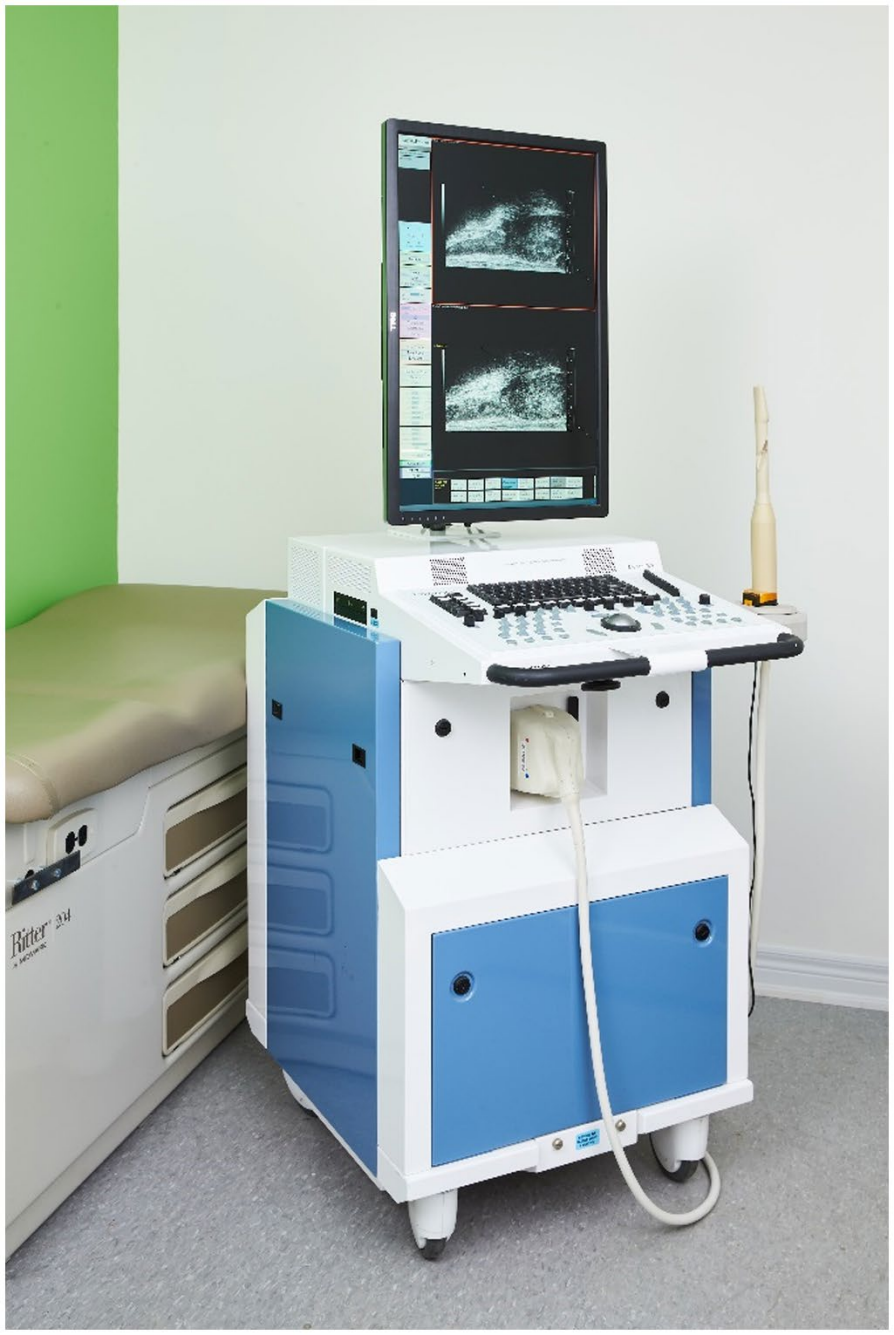
First‐generation ExactVu micro‐ultrasound system used in this study

The protocol specified that 12‐cores be taken transrectally from each subject, with each core taken either systematically or from an ultrasound target near the systematic position (and taken in lieu of the systematic core from that area); full cores were to be taken from the right and left apex, mid‐gland, and base, both laterally and medially, focusing on the peripheral zones as per standard biopsy technique. Anterior aspects of the gland were not systematically targeted. No additional cores were permitted for targets to avoid an information bias due to increased number of samples from those subjects. Periprostatic lidocaine was used as local anesthesia in all cases. The conv‐US arm exclusively employed various commercial end‐fire transducers and systems (see Supplementary Section for a list). The micro‐US arm used a specific and novel side‐fire transducer (EV29L, Exact Imaging Inc.) along with the first‐generation ExactVu^TM^ micro‐US system (Figure 1). The side‐fire transducer configuration was new to most of the investigators in the trial and some prior literature suggests a systematic sampling deficit with such a probe configuration.^16^, ^17^ This topic is addressed in more detail in the Supplementary section.

### 2.6 Image interpretation/target identification

**FIGURE 2:**
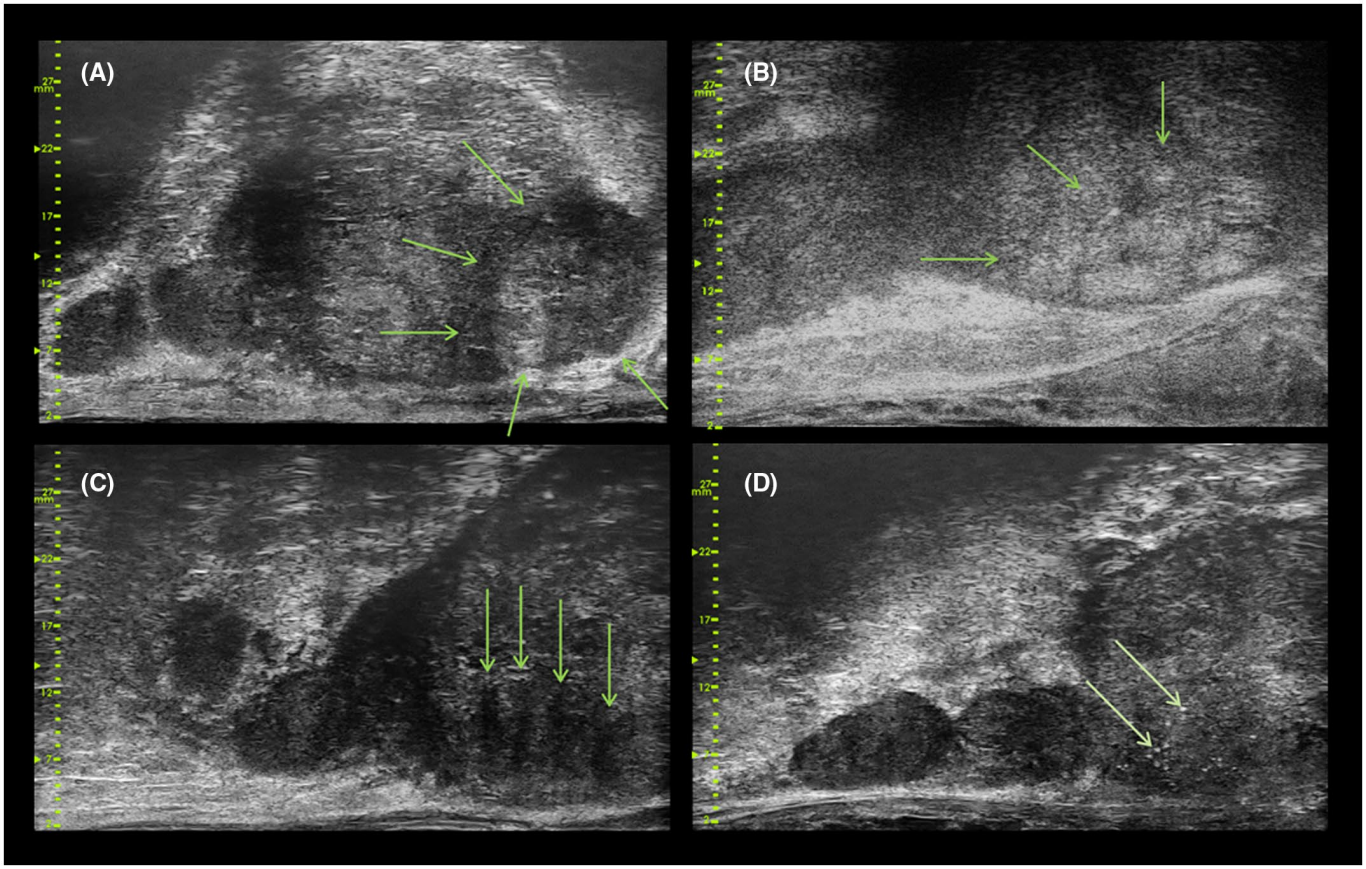
Examples of micro‐ultrasound guided biopsy targets. (A) Apex lateral mixed‐echo PRI‐MUS 5 lesion. Biopsy of this area confirmed Gleason Sum 7 (4+3) cancer in 80% of the core. (B) Mid‐prostate posterior PRI‐MUS 4 “cauliflower” patterned lesion. Biopsy of this area confirmed Gleason Sum 8 disease. (C) Base PRI‐MUS 5 irregular shadowing confirmed as Gleason Sum 7 (4+3) cancer on biopsy with 95% core involvement. (D) Base PRI‐MUS 4 bright echo pattern confirmed as Gleason Sum 7 (3+4) disease

Targets in the initial portion of the trial included areas that appeared hypoechoic and/or demonstrated gross capsular abnormalities, in both the micro‐US and conv‐US arms. In the first portion of the trial investigators were not given separate guidance on target identification using micro‐US, despite the fact that micro‐US’s 300% increase in spatial resolution provides a very different view of prostate tissue,^15^ as there was no image library from which to draw. Images and 5‐second cine‐loops were thus taken at the time of each biopsy needle and stored for subsequent image analysis. In the second portion of the trial, training was provided on the newly developed PRI‐MUS™ protocol for micro‐US target identification.^15^ Investigators were subsequently instructed to target PRI‐MUS 3, 4, and 5 areas (on a 1‐5 suspicion scale, with 5 being the most suspicious)—for examples see Figure 2.

## 3 RESULTS

### 3.1 Demographics

**FIGURE 3:**
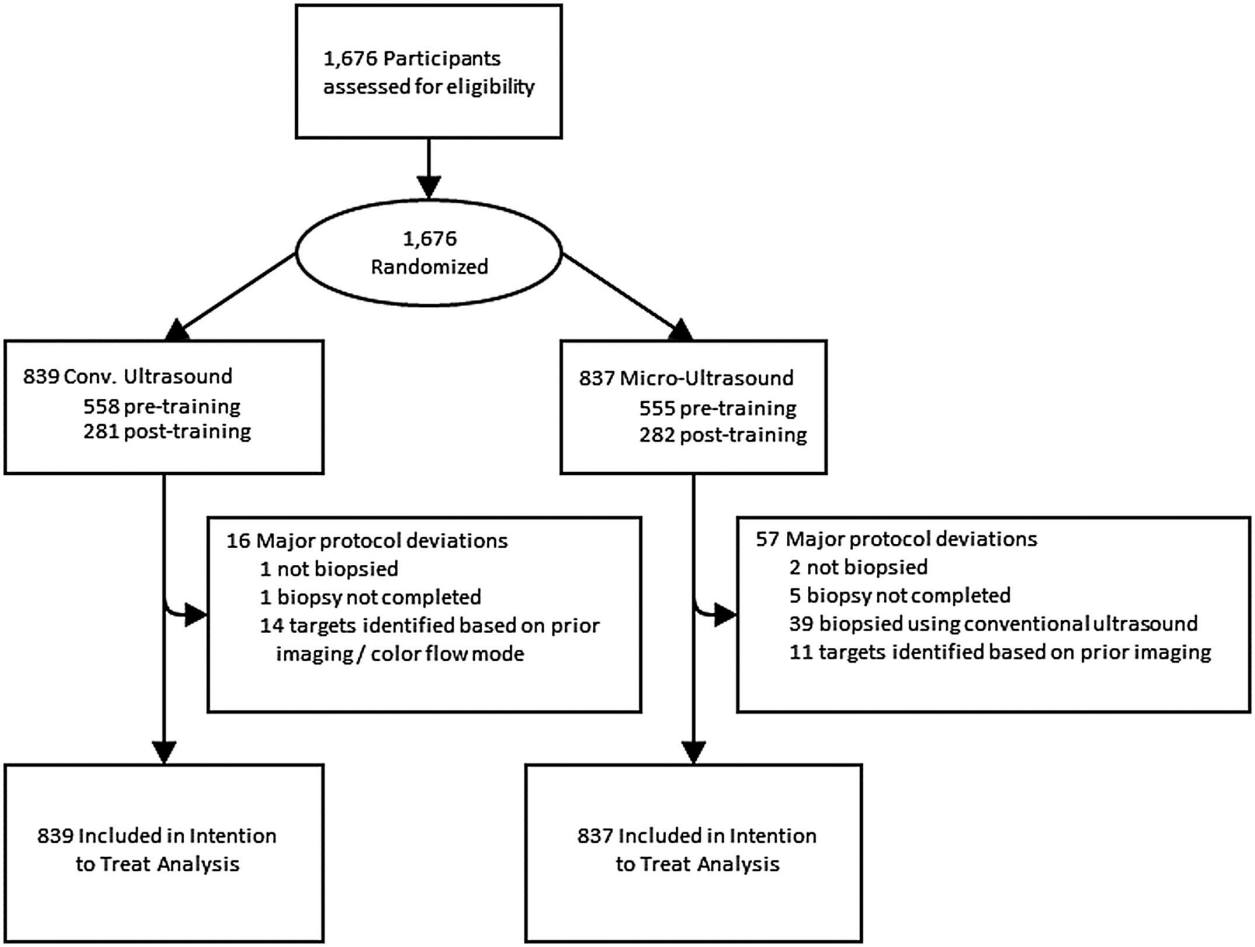
Study participant flow. 1676 subjects were enrolled and randomized

A total of 1676 subjects were randomized, 839 to Conv‐US and 837 to Micro‐US; 3 were not biopsied (1 conv‐US and 2 micro‐US), the biopsy was not successfully completed in 6 (1 conv‐US and 5 micro‐US), and all other subjects received a complete 12‐core biopsy (Figure 3). Thirty‐nine subjects in the micro‐US arm did not receive the randomized biopsy strategy and instead were biopsied using conv‐US. These included patients with very large prostates who would have been excluded had their prostate volume been known ahead of time (8), subjects who were unable to tolerate the prototype transducer (15), or a result of technical difficulties with the prototype ultrasound device for example, software crashes in the first‐generation system that have since been corrected (8). No major differences in risk factors were observed between the micro‐US and conv‐US groups (Table 1).

**TABLE 1:** Study demographics

	Overall	Micro‐ultrasound	Conventional ultrasound
Total enrolled	1676	837	839
Age (median + IQR)	63	63 [57‐68]	63 [56‐68]
PSA (median + IQR)	6.0	6.0 [4.1‐8.4]	6.0 [4.3‐8.1]
Family history of PCa	22.9%	21.5%	24.2%
Positive DRE	21.2%	21.0%	21.4%
PCPT risk score	44% [38‐52]	44% [38‐52]	44% [37‐52]

### 3.2 Complications

There were three episodes of urosepsis/systemic infection in each arm of the study for an overall infectious complication rate of 0.4% (Clavien grade 2). These were all successfully treated with intravenous antibiotics. There were no Clavien grade 3 or higher complications in either study arm.

### 3.3 Primary outcome—Detection rate of csPCa

The per‐patient detection rates of csPCa did not differ between transrectal micro‐US and conv‐US arms (34.6% vs 36.6%, respectively, *P* = .21). Cancer detection per arm and by Gleason grade is noted in Table 2. Notably, the detection rate of clinically *in*significant cancer did not differ between the two arms either (14.9% micro‐US vs 16.9% conv‐US). Neither technology was statistically more effective at diagnosing csPCa at smaller (≤40cc) or larger (>40cc) gland volumes (*P* > .05).

**TABLE 2:** Patient‐level outcome measures

	Overall	Micro‐US (%)	Conv‐US (%)	
N	1676	837	839	
Any PCa	864	415 (49.6%)	449 (53.5%)	*P* = .05
csPCa	597	290 (34.6%)	307 (36.6%)	*P* = .21
Biopsy Gleason score				One‐way ANOVA *P* = .435
6	298	144 (17.2%)	154 (18.4%)	
7 = 3 + 4	252	123 (14.7%)	129 (15.4%)	
7 = 4 + 3	147	71 (8.5%)	76 (9.1%)	
8	103	53 (6.3%)	50 (6.0%)	
9	60	23 (2.7%)	37 (4.4%)	
10	4	1 (0.1%)	3 (0.4%)	

Post hoc analyses showed that significant (and unexpected) prostate apical undersampling was noted in the micro‐US arm of upon review of biopsy cine‐loops, which likely reduced detection of csPCa in the micro‐US arm (see Supplement). The technique to properly sample the peripheral zone apical horn with side‐fire transducers is well‐established^16^, ^17^ and not thought to represent a true impediment to cancer detection using such systems.

### 3.4 Secondary outcomes—Mid‐trial retraining and per‐core performance characteristics

Mid‐trial retraining on micro‐US image interpretation in the latter portion of the study was associated with an increase in csPCa detection rate in both the micro‐US and conv‐US arms, from 32.4% to 39.0% (micro‐US arm) and from 39.0% to 39.5% (conv‐US arm); this change was only significant in the micro‐US arm (*P* < .03, Table 3). With retraining, the overall rate of csPCa detection in both arms was comparable, but more cancers were found through targeted biopsy in the micro‐US arm and fewer through systematic biopsy, while the opposite was true in the LR arm (data not shown). Post hoc analyses were performed to better understand why systematic biopsy PCa detection rates were lower in the micro‐US arm (and are presented in the Supplement). There were no differences in Table 1 patient demographics between the pre‐ and post‐training cohorts (data not shown).

**TABLE 3:** Effect of mid‐trial training. A significant improvement in micro‐US detection rate was seen after training on side‐fire micro‐US biopsy technique and image interpretation using PRI‐MUS. A smaller increase was also seen in the conv‐US arm (not significant) potentially resulting from increased focus on image optimization and interpretation

Arm		Subjects	csPCa	Detection rate	*P*‐value
Micro‐Ultrasound	Pre‐training	555	180	32.4%	<.03
	Post‐training	282	110	39.0%	
Conventional Ultrasound	Pre‐training	558	196	35.5%	.1
	Post‐training	281	111	39.5%	

Prospective validation of the PRI‐MUS lesion scoring system after retraining outperformed the retrospective validation done on the first portion of the data, demonstrating an AUC = 0.67 (*P* < .01 vs chance). This was a clear improvement over the near chance‐level targeting AUC in the pre‐training phase of the study (AUC = 0.55). The sensitivity, specificity, and per‐biopsy core csPCa detection rates were calculated before and after mid‐trial retraining (Table 4). Micro‐US after retraining was the most sensitive modality (60.8%), but the least specific (63.2%).

**TABLE 4:**
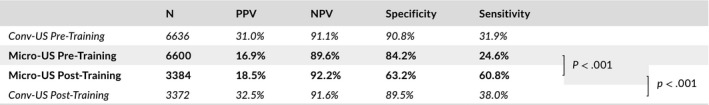
Per‐biopsy core statistics and effect of mid‐trial training. Significant improvements in sensitivity were seen post training in the micro‐US arm of the study. There was also a significant improvement in sensitivity noted between the post‐training micro‐US and post‐training conv‐US arms

**FIGURE 4:**
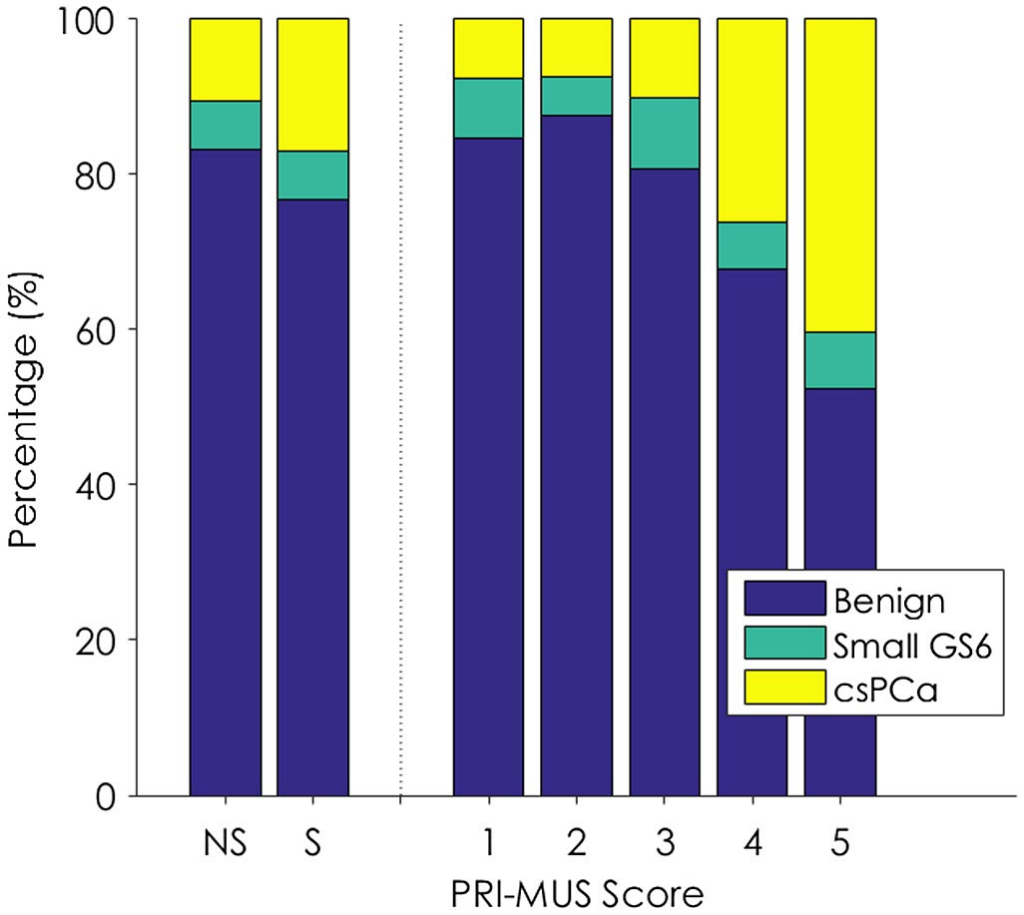
Improved risk stratification in the micro‐US arm on a per‐core level pre‐ and post‐training on PRI‐MUS. Modest difference is observed between the non‐suspicious (NS) and suspicious (S) samples taken prior to training, while clear risk stratification is evident after training with both rate of PCa and grade of PCa increasing with PRI‐MUS score. GS = Gleason score, “Small GS6” reflects no core with 50% or greater cancer

Both rate of PCa and grade of PCa increased with increasing PRI‐MUS score over the post‐training phase of the study (Figure 4). This is contrasted with the modest difference observed between the non‐suspicious and suspicious labeling performed prior to training.

## 4 DISCUSSION

This trial represents the first randomized study comparing high‐resolution, high‐frequency micro‐US with conv‐US for TRUS‐mediated prostate cancer detection. First‐generation micro‐US without image interpretation training showed no improvement in the detection of csPCa compared to conv‐US. However, this trial allowed for the creation and introduction of PRI‐MUS, a prostate cancer risk stratification protocol for real‐time interpretation of prostate images on micro‐US.^15^ A better understanding of what prostate cancers look like on micro‐US was no doubt responsible for the significant improvements in sensitivity noted in the micro‐US after mid‐trial PRI‐MUS training, though at the cost of some specificity. Importantly, there was no increase in the detection of clinically *in*significant cancer when micro‐US was used, despite higher resolution and initial inexperience with the technology.

The novel technology of micro‐US TRUS, which uses frequencies of 17‐29 MHz, was initially applied to prostate imaging in a pilot study of 25 men with known prostate cancer who were subjected to both transrectal conv‐US and micro‐US prior to radical prostatectomy.^13^ Radiologic‐pathologic correlation was performed after radical prostatectomy, and significant improvements in csPCa detection performance characteristics were found using micro‐US. These findings were enough to launch the present trial, despite there not having enough data to comprehensively characterize the various changes in echotexture appreciable on micro‐US that correlated with prostate cancer. Since the trial presented here, other groups have reported favorable single‐center and multicenter results using the second‐generation micro‐US technology. These include analysis of diagnostic value,^18^ detection rate through targeted sampling,^19^ case studies on screening and focal therapy,^20^, ^21^ prospective validation of PRI‐MUS,^22^ use of MRI/Micro‐US fusion,^23^ and comparison to mpMRI.^24^, ^25^

PRI‐MUS is in some ways comparable to PI‐RADS for MRI in use of a 1‐5 scale for increasing suspicion of cancer on biopsy, but unlike PI‐RADS it is assessed in real‐time. Increasing PRI‐MUS values were associated with increasing risk of csPCa. Unlike PI‐RADS, PRI‐MUS is not multiparametric, though in the future it may be enhanced by additional modes, including Doppler, elastography, and/or contrast enhancement. Other investigators are in fact adding such modalities in a multiparametric fashion to conv‐US such as in the CADMUS trial,^26^ where a multiparametric look at the prostate will be performed using elastography and contrast, with images analyzed at a later date and cognitive targeting of areas deemed suspicious.

Limitations of the trial included unexpectedly poor sampling of the prostate apex with the first‐generation micro‐US probe. This was likely a technical issue with probe design, as its flared base and width as well as angled biopsy channel did not always allow for easy access to the apex (especially in men with larger prostates), as well as due to lack of familiarity with side‐fire probes. Based on historical literature, such sub‐optimal systematic biopsies reduce the per‐patient detection rate of csPCa by approximately 13%^16^, ^17^; a subgroup was created to investigate the effect of these apical samples and is described in the Supplementary material. These technical challenges have been addressed by the manufacturer and data gathered using the commercially available second‐generation micro‐US system have shown significantly improved results.^24^, ^25^, ^27^ Other limitations included targeting: only 1 core/target was allowed, unlike the 3‐4 cores used during MRI‐fusion biopsy.^3^, ^28^ This likely resulted in a bias against csPCa detection on targeted samples for micro‐US since it detected far more targets than conv‐US (1907 vs 1252, *P* < .001). In addition, PRI‐MUS 3 lesions were targeted per study design though these are known to be cancerous <50% of the time^15^—this information bias decreases micro‐US specificity relative to conv‐US. This study also did not assess the learning curve with micro‐US after initial PRI‐MUS training, which may be relevant as training was done in the latter half of the study. Finally, the trial was agnostic to MRI, since it was not easily or consistently reimbursable in North America during the years of this study.

## 5 CONCLUSIONS

Micro‐US holds promise as a real‐time prostate imaging and TRUS‐biopsy targeting modality. While this trial of first‐generation micro‐US did not show improvements in csPCa detection comparing micro‐US to conv‐US, image analyses done during the trial resulted in the development of a risk stratification protocol for prostatic imaging (PRI‐MUS), which, when taught to the investigators mid‐trial, resulted in significant improvements in micro‐US cancer detection and sensitivity. Recently published data using PRI‐MUS show significantly higher cancer detection rates, suggesting that experience with micro‐US is important for it to reach its potential as an aid to prostate cancer diagnosis and lesion targeting.

## CONFLICT OF INTEREST

Dr. Pavlovich reports grants and personal fees from Exact Imaging during the conduct of the study.

## Supporting information

Supplementary Material
