# Supplementary material for: A multi‐institutional randomized controlled trial comparing first‐generation transrectal high‐resolution micro‐ultrasound with conventional frequency transrectal ultrasound for prostate biopsy
Source: BJUI Compass. 2020 Nov 28;2(2):126–33. doi: 10.1002/bco2.59 (PMC8988781; doi:10.1002/bco2.59)
Supplement: Supplementary file 1 — Supplementary Material [file BCO2-2-126-s001.docx]

**Supplementary Material – Conventional Ultrasound Systems Used**

| **Centre** | **System** | | | |
| --- | --- | --- | --- | --- |
| Johns Hopkins | Manufacturer | Hitachi Aloka Medical Ltd. | | |
|  | System Model | HI Vision Avius | | |
|  | Year of Manufacture | 2013 | | |
|  | Transducer Model | Endocavity Ultrasound Probe EUP-V53W | | |
| Prostate Cancer Centre | Manufacturer | Toshiba x2 | | |
|  | System Model | Xario x2 | | |
|  | Year of Manufacture | 2006 | | |
|  | Transducer Model | Toshiba PVT-770RT | | |
| CRCEO | Manufacturer | Toshiba | | |
|  | System Model | SSA-790A | | |
|  | Year of Manufacture | 2011 | | |
|  | Transducer Model | PVT-66VT | | |
| Princess Margaret Cancer Centre | Manufacturer | Phillips | | |
|  | System Model | IU22 | | |
|  | Year of Manufacture | 2007 | | |
|  | Transducer Model | C9-5ec | | |
| Urology of Virginia |  | System # 1 | System # 2 | System # 3 |
|  | Manufacturer | Phillips | GE | BK Medical |
|  | System Model | HD15 | Logic P5 | Falcon Ultrasound Scanner Type 2101 |
|  | Year of Manufacture | 2013 | 2010 | 2004 |
|  | Transducer Model | BP10-5ec | BE 9CS | BCFM Mode Type 8667 |

**Supplementary Material – Effect of Apical Horn Undersampling**

Retrospective review of the data revealed systematic under-sampling of the prostate apex, particularly the apical horn where the biopsy needle often captured very little peripheral zone tissue due to basal placement of the needle and angle of entry. Figure S1 shows two examples of apical horn biopsy locations from the study. Measurements of distance from the Apex to the needle guide were made manually using custom software designed in MATLAB (Mathworks Inc.). To avoid bias, all measurements were made while blinded to the pathological outcome of the sample.


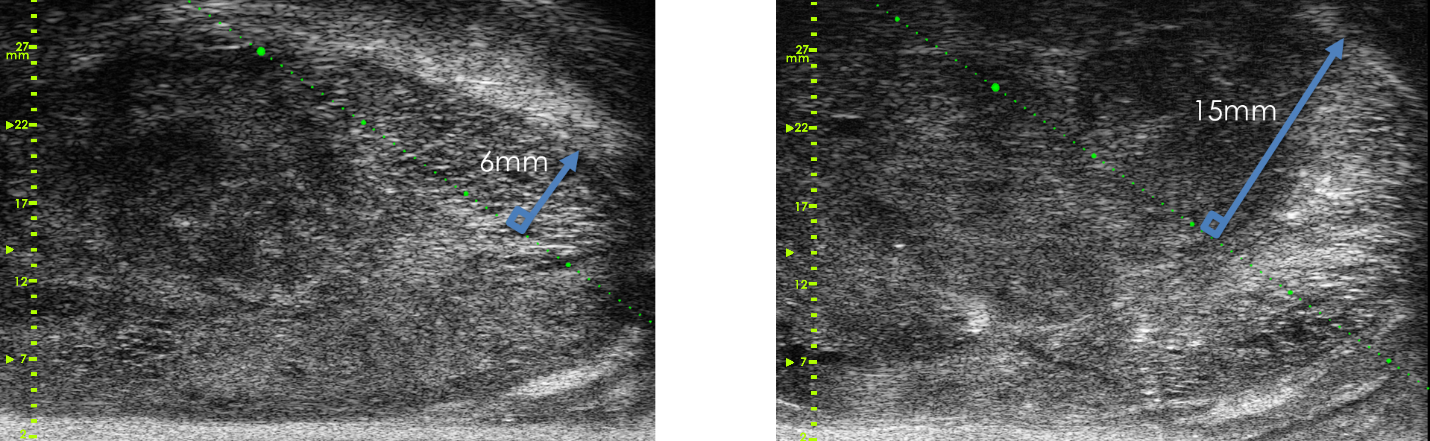


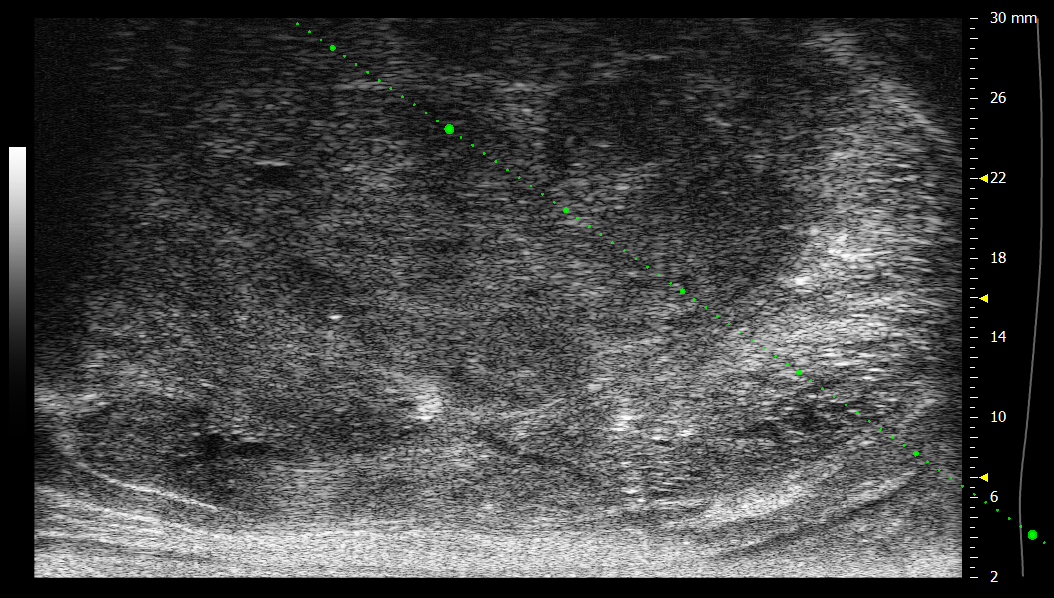


15mm

Figure S1 – Measurements were made on each image just before biopsy needle penetration. All measurements were made from the biopsy needle guide perpendicularly to the prostate capsule. The image on the left illustrates a good sample, containing nearly all peripheral zone tissue from the apical horn, while the image on the right misses a large amount of peripheral zone tissue in the anterior portion of the horn, thereby under-sampling the apical biopsy.

A threshold of 9.5mm was selected based on the first half of the measurements analyzed and was applied to the final dataset. This per-protocol dataset which excludes major protocol violations in both arms, as well as cases with gross apical horn undersampling in the micro-ultrasound arm demonstrated a significant benefit for micro-ultrasound in csPCa detection with 125/286 (43.7%) csPCa in the micro-ultrasound arm vs. 301/823 (36.6%) csPCa in the conventional ultrasound arm (p=0.02).

Figure S2 shows the effect of the apical horn measurement threshold trading off number of subjects included in the Per Protocol group vs. the Improvement over the conventional arm of the study. Note that the improvement level is significant over a very wide range of thresholds, showing an almost linear trend within the 7-12mm region. In hindsight, the selected threshold was not optimal, with higher improvement levels (up to 40% higher detection rate compared to conventional ultrasound) at 7mm. This suggests that detection rates with micro-ultrasound may be higher than predicted by this study when proper apex sampling technique is applied. Similarly, with a less stringent threshold over 400/837 subjects could have been included while maintaining statistical significance of the superiority result.

As a second independent verification, a post-hoc analysis was performed eliminating all apical samples from the database under the hypothesis that if these samples were poorly performed with micro-ultrasound ignoring them would cause a smaller decline in detection rate. Eliminating these samples cause the micro-ultrasound csPCa detection rate to drop from 290/837 (34.7%) to 262/837 (31.3%) for a decline of 3.4%. Similarly, the csPCa detection rate for conventional ultrasound dropped from 307/839 (36.6%) to 258/839 (30.8%) for a decline of 5.8% (1.7-fold higher). While this basic analysis ignores any effect from targeted sampling, it does support the conclusion that apical sampling was not effectively performed in many of the micro-ultrasound cases during this study.

Figure S2 – Trade-off between number of subjects included in Per Protocol group and improvement over conventional ultrasound. Note that curve is relatively smooth, particularly within the mid-range of threshold values between 7 and 12mm. The green highlighted area indicates a p-value <0.01 for improvement over conventional ultrasound, while the yellow area has a p-value <0.05. This data indicates that precise choice of threshold would not have significantly altered the conclusions of the study, as a wide range of clinically reasonable choices provide sufficient p-values to demonstrate superiority of micro-ultrasound to conventional ultrasound.

This study was conducted with a 1^st^ generation micro-ultrasound system, that is somewhat different than the current 2^nd^ generation commercial version of the micro-ultrasound system in current clinical use. The design and ergonomics of the 2^nd^ generation micro-ultrasound transducer have been significantly modified to support easier apical horn biopsy technique, which is demonstrated below in Figure S3.


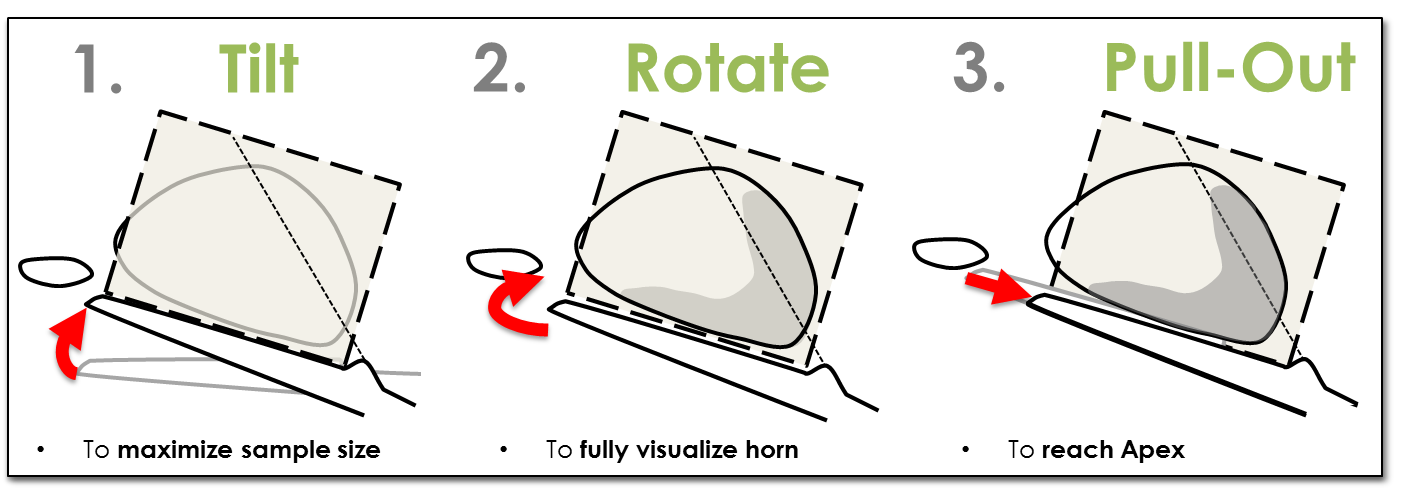


Figure S3 – Optimal apical horn biopsy technique. In order to correctly sample the apical horn with a micro-ultrasound side-fire transducer, the transducer must be tilted as shown in step 1. This compresses the prostate, particularly at the base and aligns the needle guide with the apical capsule wall. Step 2 is to rotate the transducer to visualize the point of the apical horn, and then step 3 is to pull out slightly to align the needle guide so that as much of the horn tissue is sampled as possible.
